# The effect of endurance training and testosterone supplementation on the expression of blood spinal cord barrier proteins in rats

**DOI:** 10.1371/journal.pone.0211818

**Published:** 2019-02-11

**Authors:** Katarzyna Nierwińska, Marta Nowacka-Chmielewska, Jacek Bernacki, Sławomir Jagsz, Małgorzata Chalimoniuk, Józef Langfort, Andrzej Małecki

**Affiliations:** 1 Department of Physiology, The Jerzy Kukuczka Academy of Physical Education, Katowice, Poland; 2 Laboratory of Molecular Biology, The Jerzy Kukuczka Academy of Physical Education, Katowice, Poland; 3 Department of Pharmacology, Medical University of Silesia, Katowice, Poland; 4 Department of Biochemistry, The Jerzy Kukuczka Academy of Physical Education, Katowice, Poland; 5 Department of Tourism and Health in Biala Podlaska, Piłsudski University of Physical Education in Warsaw, Warsaw, Poland; 6 Department of Sports Training, The Jerzy Kukuczka Academy of Physical Education in Katowice, Katowice, Poland; University of Kentucky Medical Center, UNITED STATES

## Abstract

The present study aimed to estimate the effect of endurance training, two doses of testosterone, and the combination of these stimuli on the level of the endothelial proteins claudin, occludin, JAM-1, VE-cadherin, ZO-1, ZO-2, and P-glycoprotein in rat spinal cords. Adult male Wistar rats were trained using a motor-driven treadmill for 6 weeks (40–60 min, 5 times per week) and/or were treated for 6 weeks with two doses of testosterone (i.m.; 8 mg/kg or 80 mg/kg body weight). Spinal cords were collected 48 hours after the last training cycle and stored at -80°C. The levels of selected proteins in whole tissue lysates of the spinal cord were measured by western blot. Testosterone-treated trained rats had significantly lower claudin levels than vehicle-treated trained rats. High doses of testosterone resulted in a significant decrease in claudin-5 in untrained rats compared to the control group. Both doses of testosterone significantly reduced occludin levels compared to those in vehicle-treated untrained rats. The JAM-1 level in the spinal cords of both trained and untrained animals receiving testosterone was decreased in a dose-dependent manner. The JAM-1 level in the trained group treated with high doses of testosterone was significantly higher than that in the untrained rats treated with 80 mg/kg of testosterone. VE-cadherin levels were decreased in all groups receiving testosterone regardless of endurance training and were also diminished in the vehicle-treated group compared to the control group. Testosterone treatment did not exert a significant effect on ZO-1 protein levels. Testosterone and/or training had no significant effects on ZO-2 protein levels in the rat spinal cords. Endurance training increased P-glycoprotein levels in the rat spinal cords. The results suggest that an excessive supply of testosterone may adversely impact the expression of endothelial proteins in the central nervous system, which, in turn, may affect the blood-brain barrier function.

## Introduction

The blood-brain barrier (BBB) and the blood-spinal cord barrier (BSCB) play important roles in protecting the central nervous system (CNS) from the influence of external factors such as hormones and xenobiotics. Alterations to the barrier integrity are directly involved in the trafficking of inflammatory cells into the brain and in the development of neuroinflammatory responses. The BBB and the BSCBs are not only physical barriers, but they also control influx and efflux transport as well as contain the drug-metabolizing enzymes. Certain bioactive substances, including toxic substances, may lead to disorders affecting the function of the BBB and BSCB. Together, the BBB and BSCB are important conduits and interfaces in humoral-based communication between the CNS and the rest of the body [1].

The barrier function of the spinal cord capillaries is based on a specialized system of non-fenestrated endothelial cells and their accessory structures, including the basement membrane, pericytes, and astrocytic end-feet processes. These structures are responsible for the regulatory and protective functions of the BSCB. There are several structural and functional differences between the BBB and the BSCB; these include the presence of glycogen deposits in the spinal cord micro-vessels, which may cause variations in glucose uptake and metabolism [2].

Paracellular permeation in both the BBB and BSCB is regulated by the multi-protein tight-junction complex. The permeation properties of this complex are determined by the protein composition [3]. The decreased expression of the tight-junction proteins, ZO-1 and occludin, have been observed in cultured microvascular endothelial cells from the murine spinal cord compared to brain microvasculature endothelial cells. Similarly, the expression of adherence junction proteins was also decreased. Furthermore, VE-cadherin and β-catenin were reduced in both the spinal cord microvessels and cultured spinal cord endothelial cells [4]. Ge et al. (2006) demonstrated an insignificant decrease of P-glycoprotein in spinal cord endothelial cells, with no differences between the von Willebrand factor and PECAM-1 expression between brain and spinal cord-derived cultures [4]. The decreased expression of both tight junction (ZO-1 and occludin) and adherence junction proteins was also observed in cultured endothelial microvascular cells from the murine spinal cord compared to brain microvasculature endothelial cells, which may serve as an explanation for the increased permeability of the BSCB compared to the BBB [4]. Following treatment with interferons α and γ (IFNα and IFNγ) and tumor necrosis factor α (TNFα), the BSCB permeability was higher compared to that of the BBB [4]. Permeability differences have also been observed between the spinal cord regions which may be related to the variability of spinal cord functions, in turn, suggesting that the BSCB should be studied in parallel with the BBB [5,6].

Sex steroid hormones affect spinal cord blood vessels, as well as the cells of which they are composed; these effects are, for the most part, elicited through binding of specific receptors [7]. Androgen receptors (ARs) in the spinal cord are located predominantly in the cell bodies of the motoneurons, in the ventral horns, and in the sensory parts of the dorsal roots [8]. In males, the main area for the expression of ARs in the spinal cord is the spinal nuclei of the bulbocavernosus (SNB), which exhibits sexual dimorphism [9]. The level of ARs in the spinal cord increases in animals treated with androgens [10].

Substitutive doses of testosterone are used in humans in certain circumstances, e.g. in the still controversial hormone-replacement therapy [11]. There are suggestive data indicating that androgens are independent factors in the development of atherosclerosis and its prevalence in men [11, 12, 13]. Moreover, it has been shown that the use of androgens may be associated with coronary disease in athletes and in transsexual female-to-male individuals [11]. The decrease of testosterone levels in humans may occur as a result of aging in men or bilateral ovariectomy in women; such circumstances are associated with hypertension, diabetes, and atherosclerosis [13]. Therefore, replacement therapy may prove beneficial, mostly through the restoration of hemostatic functions and anti-atherosclerotic activity [11]. Moreover, physical training is often combined with anabolic-androgenic steroid (AAS) abuse, which may lead to a number of disorders. Such relatively common androgen abuse by athletes or body builders may be simulated in animal studies by using higher, sometimes referred to as supraphysiological, doses of testosterone. Additionally, it has been proposed that stress related to physical exercise may impair the functioning of the blood-brain barrier [14, 15, 16, 17, 18].

Considering the lack of data on the effect of testosterone in combination with physical training on the blood-brain barrier, we have decided to convey the experiments on rats subjected to endurance training on a treadmill. Two doses of testosterone were employed to estimate the effective modes of the administration of this drug in the current experimental approach. The doses of testosterone were chosen according to a previous study published by Sadowska et al., 2011 [19]. We had two hypotheses in terms of the current study: 1) that endurance training exerts certain effects on the BSCB in rats, and 2) that higher doses of testosterone have detrimental effects on the blood-spinal cord barrier and may interfere with the effects of training.

## Materials and methods

### Laboratory animals

All experiments were conducted on 6-week-old male Wistar rats (M. Mossakowski, Polish Academy of Sciences, Institute of Experimental and Clinical Medicine, Warsaw, Poland) weighing 100–120 g. The animals were kept under standard conditions with a stable temperature of 22–24°C, with a relative humidity of 55%–60%, and under a 12-hour day/night-cycle (lights on at 7:00 a.m.). The animals were fed ad libitum with a standard rodent pellet diet with constant free access to water. All experiments were carried out in accordance with the guidelines of the European Ethical Standards (2010/63/EU) and were approved by the Local Ethics Committee for the Care and Use of Laboratory Animals (resolution number 64/2009, Warsaw, Poland).

### Experimental design

Prior to the start of the experiment, all rats were run-tested on a motorized rodent treadmill (3, 5, and 9 min with 15-min breaks) for 3 successive days in order to habituate them to the training environment and to eliminate those rats that were unwilling to run. The selected rats were randomized between the following research groups:

- **Veh** (n = 20): untrained animals (the control group), receiving sesame oil *im*.
- **Tr** (n = 20): trained rats, receiving sesame oil *im*.
- **T [8 mg/kg]** (n = 19): untrained animals receiving *im*. testosterone propionate at a low dose of 8 mg/kg,
- **TrT [8 mg/kg]** (n = 19): trained animals receiving *im*. testosterone propionate at a low dose of 8 mg/kg,
- **T [80 mg/kg]** (n = 20): untrained animals receiving *im*. testosterone propionate at a high dose of 80 mg/kg,
- **TrT [80 mg/kg]** (n = 20): trained animals receiving *im*. testosterone propionate at a high dose of 80 mg/kg.

### Testosterone administration

The stock TP solution (*Testosteronum propionicum*; Jelfa, Poland) was diluted with sesame oil as necessary before injecting intramuscularly, once per week for 6 weeks, alternating between the right and left hindlimb. The TP dosage was based on the protocol used by Sadowska et al. [19]. The solution of testosterone in sesame oil was prepared immediately before administration. The control groups were given the same volume of sesame oil alone according to the same schedule.

### Endurance training

The rats scheduled for endurance training were exercised on a rodent treadmill (at a 0° inclination) 5 days per week for 6 weeks. The treadmill speed was 16 m/min during the first week, increased by 4 m/min weekly over the next 3 weeks, and kept at 28 m/min for the remaining training sessions. The session duration started each week at 40 min/day and was increased by 5 min daily during the first 4 weeks; during the last 2 weeks, the rats ran daily for 1 hour.

### Preparation of spinal cord tissue homogenate

Two days after the completion of the training, the rats were euthanized by decapitation and their spinal cords were isolated. Next, the spinal cords were manually homogenized at 4°C in a glass dounce homogenizer (thirteen offsets of the piston). The isolation buffer used was as follows: 1 M TRIS-HCl, 5 M NaCl, 10% SDS, 0.2 M EDTA, Igepal, PMSF 10 mg/1 ml, aprotinin 5 mg/1 ml, pepstatin 2 mg/1 ml, leupeptin 1 mg/1 ml, 0.1 M Na_3_VO_4_ and bidistilled water. The lysate was centrifuged at 4°C for 60 min at 15,000 x g. The supernatant obtained by centrifugation (the cytosolic fraction) was administered for further determinations by the western blot method. Following the measurement of protein concentration using the Bradford method, the material was stored at -80°C.

### Western blot

Equal amounts of pure protein from each sample were separated by sodium dodecyl sulfate-polyacrylamide gel electrophoresis (8% acrylamide) and transferred to a nitrocellulose membrane at 250 mA for 1.5 hours at 4°C. Nonspecific binding was blocked by an overnight incubation of the nitrocellulose membrane with 5% fat-free milk in a Tris-buffered solution at 4°C. Selected monoclonal antibodies were used as a primary antibodies (Table 1), and IgG labeled with horseradish peroxidase was used as a secondary antibody (Table 2).

**Table 1:** Primary antibodies used in western blotting.

Antibody	Species	Dilution	Supplier	Code
Claudin-5	Mouse polyclonal	1:500	Invitrogen/Zymed/ BioSource	352500
Occludin	Rabbit polyclonal	1:500	Invitrogen/Zymed/ BioSource	711500
ZO-1	Rabbit polyclonal	1:500	Invitrogen/Zymed/ BioSource	402200
ZO-2	Rabbit polyclonal	1:500	Invitrogen/Zymed/ BioSource	389100
VE-cadherin	Goat polyclonal	1:500	Santa Cruz Biotechnology	sc-6458
JAM-1	Mouse monoclonal	1:1000	Santa Cruz Biotechnology	sc-53622
P-gp	Mouse monoclonal	1:1000	Santa Cruz Biotechnology	sc-390883

**Table 2:** Secondary antibodies used in western blotting.

Antibody	Dilution	Supplier	Code
Polyclonal donkey anti-goat IgG-HRP	1:5000	Santa Cruz Biotechnology	sc-2020
Polyclonal goat anti-rabbit IgG-HRP	1:5000	Santa Cruz Biotechnology	sc-2004
Polyclonal goat anti-mouse IgG-HRP	1:2000	Santa Cruz Biotechnology	sc-2005

The chemiluminescence emitted from the luminol oxidized by the horseradish peroxidase was detected using the enhanced chemiluminescence western blotting detection system (Amersham Pharmacia Biotech, Inc., Piscataway, NJ, U.S.A.). The band densities were compared using ImageJ (National Institute of Health, USA).

### Statistical analysis

Statistical analysis was performed using GraphPad Prism, v5.01 (Graph Pad Software Inc., San Diego, CA). A normal distribution of all data sets was confirmed by the Shapiro-Wilk test (alpha = 0.05). Western blot results were analyzed using a two-way analysis of variance (ANOVA) for the following factors: training (untrained vs. trained) and treatment (vehicle vs. T8 and vehicle vs. T80), followed by Tukey’s multiple comparisons test when appropriate. The results are presented as a mean ± standard deviation (SD). Differences were considered to be statistically significant when p < 0.05.

## Results

**Fig 1:**
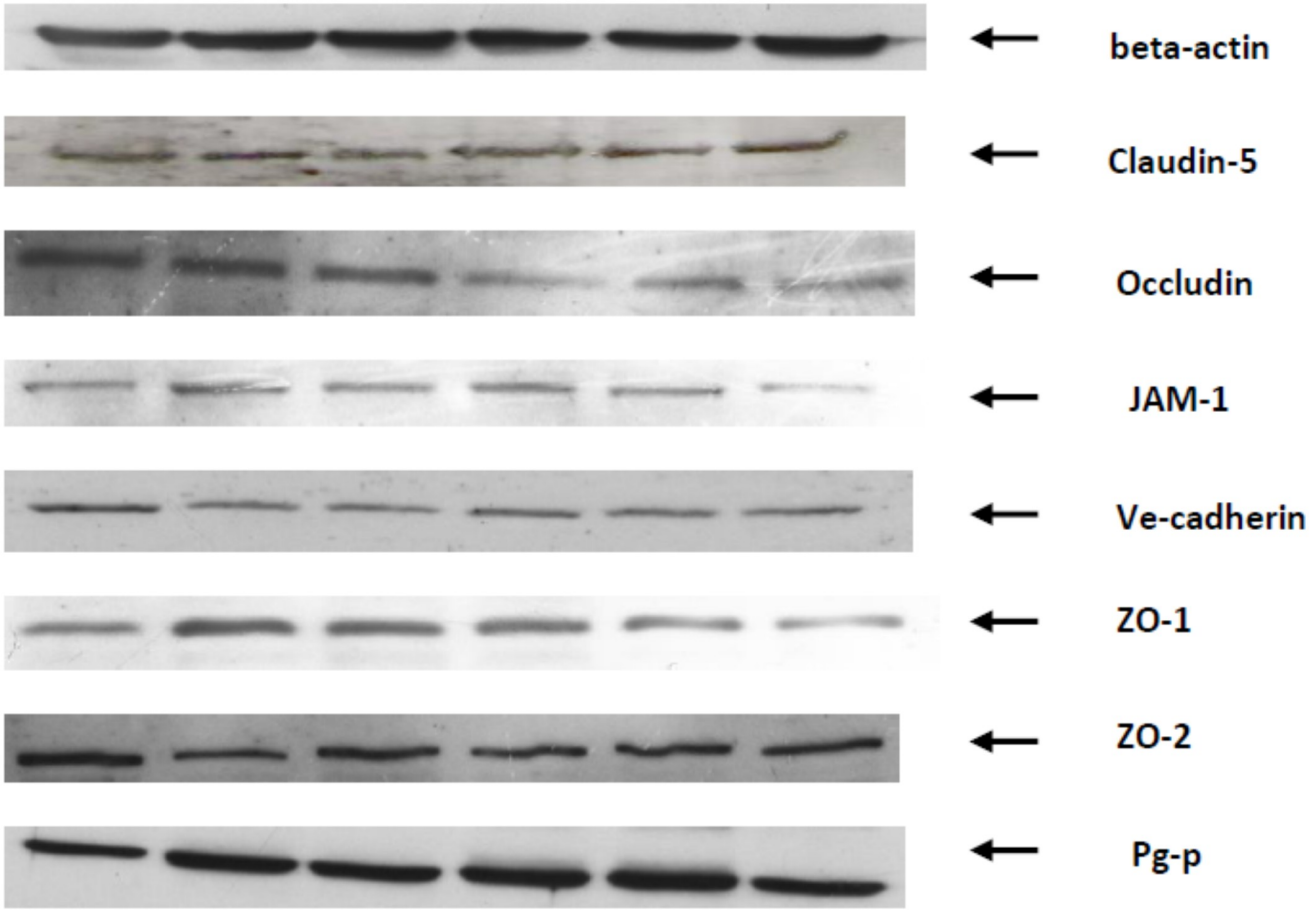
Representative western blot images of the blood-spinal cord barrier proteins of rats after endurance training and/or treatment with two doses of testosterone.

The images obtained by the western blot method are presented in the collective figure (Fig 1).

### Claudin-5

**Fig 2:**
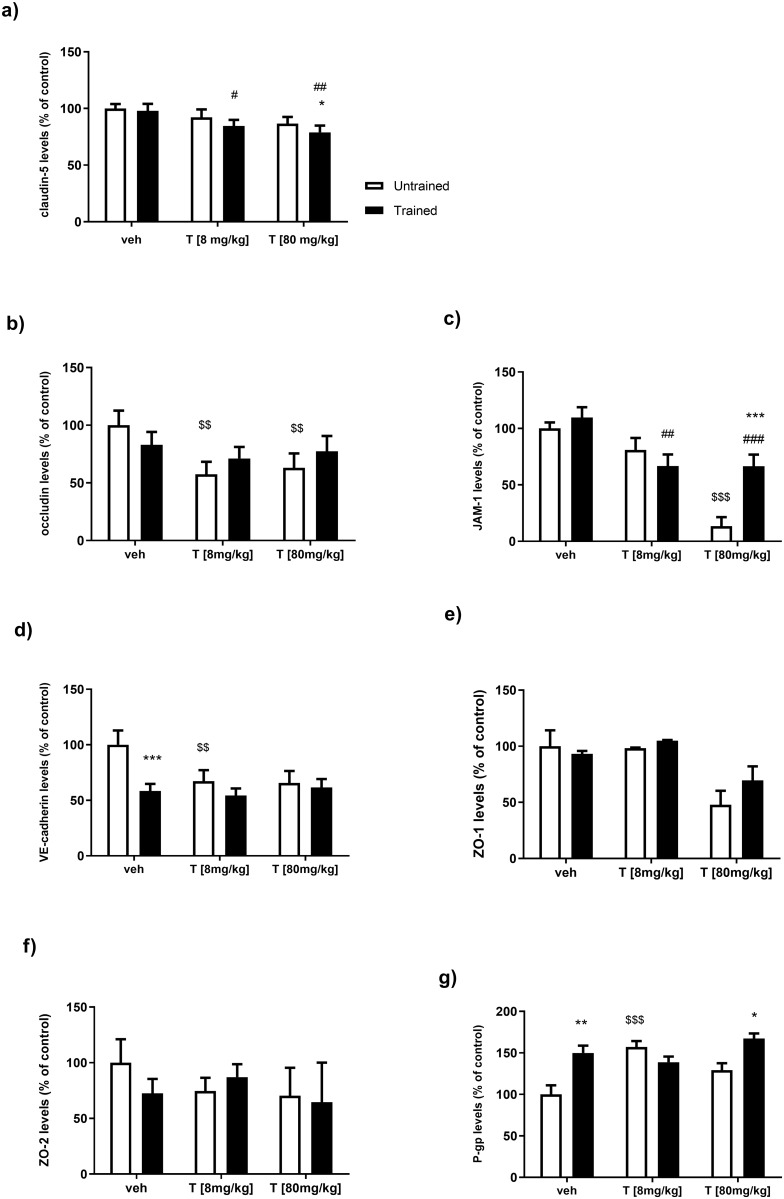
The effect of endurance training and/or testosterone administration on: a) Claudin-5 levels in the spinal cords of male rats. Values are expressed as a percentage of the control. Data were analyzed with a two-way ANOVA and post-hoc Tukey’s test. # p = 0.024 and ## p = 0.0018 vs. Tr *p = 0.025 vs. TrT [80 mg/kg]. b) Occludin levels in the spinal cords of male rats. Values are expressed as a percentage of the control. Data were analyzed with a two-way ANOVA and post-hoc Tukey’s test. $ $ p = 0.0032 (for T [8 mg/kg]) vs. Veh, $ $p = 0.0095 (for T [80 mg/kg]) vs. Veh. c) JAM-1 levels in the spinal cords of male rats. Values are expressed as a percentage of the control. Data were analyzed with a two-way ANOVA and post-hoc Tukey’s test. ##p = 0.0001, ###p < 0.0001 Tr, ***p < 0.0001 vs. T [80 mg/kg], $ $ $p < 0.0001 vs. Veh. d) VE-cadherin level in the spinal cords of male rats. Values are expressed as a percentage of control. Data were analyzed with a two-way ANOVA and post-hoc Tukey’s test. $ $p = 0.0015 vs. Veh, ***p = 0.0002 vs. Veh. e) ZO-1 levels in the spinal cords of male rats. Values are expressed as a percentage of the control. Data were analyzed with a two-way ANOVA. f) ZO-2 levels in the spinal cords of male rats. Values are expressed as a percentage of the control. Data were analyzed with a two-way ANOVA. g) P-gp level in the spinal cords of male rats. Values are expressed as a percentage of the control. Data were analyzed with a two-way ANOVA and post-hoc Tukey’s test. *p = 0.012 and **p = 0.0014 vs. Veh, $ $ $p = 0.0004 vs. Veh.

The two-way ANOVA showed significant effects of endurance training (F = 8.09, p = 0.014) and testosterone treatment (F = 21.54, p = 0.0001) on the claudin-5 content in rat spinal cords (Fig 2a). There was no significant testosterone treatment × training interaction effect (F = 0.83, p = 0.45).

A post hoc comparison revealed significantly lower levels of claudin-5 in trained animals receiving both doses of testosterone (for TrT [8 mg/kg]: p = 0.024 and for TrT [80 mg/kg]: p = 0.0018) compared to the vehicle-treated trained group. The high dose of testosterone, when used in trained rats, significantly decreased the levels of claudin-5 compared to the untrained group, which received T [80 mg/kg] (p = 0.025).

### Occludin

With regard to the levels of occludin in the spinal cord, the two-way ANOVA revealed significant effects of testosterone treatment (F = 11.34, p = 0.0017) and a significant interaction between training and testosterone treatment (F = 4.43, p = 0.036) (Fig 2b). There was no significant effect on the training factor (F = 0.56, p = 0.46).

A post hoc comparison showed that both doses of testosterone significantly reduced the levels of occludin compared those in vehicle-treated untrained rats (for T [8 mg/kg]: p = 0.0032 and for T [80 mg/kg]: p = 0.0095).

### JAM-1

The two-way ANOVA showed significant effects of endurance training and testosterone treatment on adhesion JAM-1 protein levels (F = 122.9, p < 0.001; F = 22.98, p < 0.0001, respectively), and a significant interaction between these factors (F = 34.07, p < 0.0001) (Fig 2c). There was a dose-dependent reduction in the level of the JAM-1 protein in the spinal cords of both trained and untrained animals receiving testosterone, as revealed by post-hoc analysis. The post hoc analysis showed that JAM-1 levels in the group trained and treated with high doses of testosterone were significantly higher than those in untrained rats treated with 80 mg/kg of testosterone (p < 0.0001), suggesting that endurance training has a restorative effect on JAM-1 protein levels. Post hoc comparisons also showed that both doses of testosterone significantly reduced JAM-1 levels compared to those in vehicle-treated trained rats (for TrT [8 mg/kg]: p = 0.0001, for TrT [80 mg/kg]: p < 0.0001). The post hoc analysis indicated that a high dose of testosterone in the group of untrained rats significantly lowered the levels of JAM-1 protein compared to those in vehicle-untrained rats (for T [80 mg/kg]: p < 0.0001).

### VE-cadherin

The two-way ANOVA showed significant effects of endurance training and testosterone treatment factors on VE-cadherin protein levels (F = 32.26, p = 0.0001; F = 11.16, p = 0.0018, respectively), as well as a significant T treatment x training interaction effect (F = 10.83, p = 0.0021) (Fig 2d). The level of this transmembrane glycoprotein of the tight junctions decreased in all of the groups receiving testosterone, regardless of whether or not they participated in endurance training. Training, used as a single stimuli, showed a similar effect compared to the control group. Post hoc comparisons showed a reduction in VE-cadherin levels in the Tr group compared to the Veh group (p = 0.0002); this effect was eliminated by administration of testosterone. Post hoc analysis also indicated that a dose of 8 mg/kg of testosterone in the group of untrained rats significantly lowered the level of VE-cadherin protein compared to that in vehicle-untrained rats (for T [8 mg/kg]: p < 0.0015).

### ZO-1 and ZO-2

The two-way ANOVA revealed significant effects of testosterone treatment on ZO-1 protein levels (F = 6.04, p = 0.036; Fig 2e). We observed no statistically significant effects of testosterone and/or training on ZO-2 protein levels in the rat spinal cords (Fig 2f).

### P-glycoprotein (P-gp)

The two-way ANOVA demonstrated significant effects of endurance training and testosterone (T) treatment on the level of P-gp (F = 19.72, p = 0.0008; F = 8.75, p = 0.0045, respectively, (Fig 2f), as well as significant T treatment × training interaction effects (F = 16.26, p = 0.0004). Post-hoc analysis revealed an increase in P-gp in the spinal cords of trained rats compared to the control (p = 0.0014). Similar effects were observed in trained animals, which were given a high dose of testosterone T [80 mg/kg], compared to the T [80 mg/kg] group (p = 0.012). Post hoc analysis also showed a significant increase in P-gp levels in the T [8 mg/kg] group compared to those in the Veh group (for T [8 mg/kg]; 0.0004).

## Discussion

The proper functioning of the BSCB depends upon tight junctions which are specialized proteins responsible for the maintenance of a solid structure. Among the most significant, are the tight junctions (TJs) between cells (built by the proteins ZO-1, ZO-2, claudin-5, and occludin) and the adhesion junctions (constituted e.g. by VE-cadherin). The effects of AR ligands on the TJs in CNS vessels have not yet been studied [20].

Furthermore, in rodent tests, testosterone and follicle-stimulating hormone (FSH) have been previously shown to be of significant importance in the maintenance of the appropriate distribution of claudin-11 and JAM-A at the blood-testis barrier (BTB) [21]. Testosterone was also shown to stimulate steady-state levels of occludin in the Sertoli cell epithelium [22]. However, the mechanism of action whereby steroid hormones can alter the permeability of the blood-brain barrier, as well as the expression of gap and tight junction proteins, remains unclear. A lowering of the blood-brain barrier integrity correlates with an age-related decrease in the amount of sex hormones in the blood plasma during andro- or menopause [23].

The effects of sex steroids on the brain endothelium appears to depend on hormones either present in circulation or synthesized locally. The hormone concentration within the vessel walls could reach a higher level than is anticipated on the basis of its concentration in the blood. Due to the ambiguity of the local metabolism of testosterone, the final effect on the CNS vasculature may be the result of the activity of both testosterone and the more potent metabolite dihydrotestosterone (DHT) as well as estrogen. It could be hypothesized that aromatase may constitute a negative feedback loop, attenuating and limiting the influence of androgens on vessels by stimulating the vasoprotective effects of locally produced estrogen [7].

To date, a limited number of studies have evaluated the effects of androgens on cerebrovascular function. The results of these studies differ and both beneficial and detrimental effects have been observed [24]. The existing data show that testosterone modulates the protein levels of claudin-5 and ZO-1 in the BBB of male mice [25]. Another study reported that a pro-androgen sulfated steroid stimulates the expression of claudin-5 protein and tight junction formation in Sertoli cells *in vitro* [26].

The study by Sumanasekera et al. (2007) [27] demonstrated that sex hormones regulate occludin by both genomic and nongenomic mechanisms in the human vascular endothelial cells. The authors reported that a 24 hour pretreatment of human umbilical venous endothelial cells (HUVECs) with 10 nM DHT increased the expression of occludin. The correlation between endothelial permeability, occludin levels, MAPK activation, cyclooxygenases activity, and DHT activity was also demonstrated.

To determine the effect of sex hormones on the permeability of the blood-brain barrier and the expression of tight junction proteins, Wilsson et al. (2008) [28] divided 3-month-old female rats into the following groups: control, subjected to gonadectomy, and castrated; all groups received a GnRH agonist. The castration of animals caused a significant increase in the penetration of dye into the brain; however, there was no concomitant change in the expression of the cytoplasmic tight junction protein ZO-1 in the cerebral blood vessels.

In the present study, both doses of testosterone significantly decreased the levels of several TJs proteins tested, namely: occludin, JAM-1, and VE-cadherin. We were unable to demonstrate any significant effects of testosterone on either ZO-1 or ZO-2 proteins levels. It is well known that the proteins forming TJs may be disrupted or translocated into the cytoplasm. Despite measuring the level of proteins in total homogenates by western blot, we were unable to determine their exact location. Thus, the study of TJ protein expression in the whole tissue lysate of rat spinal cords alone may be considered as a limitation of the present study. One may assume that external non-barrier proteins in whole tissue lysates may affect the final results and that the analysis of TJ proteins should also be performed in the isolated microvessels fraction. Studies show that changes in the expression of TJ proteins are different to that of the endothelial cells of the CNS, such as occludin in astrocytes, neurons, and oligodendrocytes [29, 30]. It is quite possible that the actual function and status of the analyzed proteins in the endothelial cells could be more complicated than is estimated in this study.

The regulation of substance trafficking through the BBB and BSCB constitutes one of its most important roles. Several proteins are involved in this regulation, however, special attention is dedicated to P-gp, encoded by *MDR1*. P-gp is abundant in the membrane of CNS endothelial cells, where it determines the access of a variety of drugs and substances [31]. The fact that P-gp is also an important efflux pump for a number of steroid hormones, including testosterone and progesterone, may be of particular interest [1].

Several studies have shown that sex hormones, namely progesterone and estradiol, increase P-gp expression via transcriptional regulation [32]. Mutoh et al. (2006) [33] showed in human breast cancer cells that estradiol, working via ERβ, down-regulated the P-gp protein expression level via a posttranslational mechanism.

However, the effect of androgens on the expression and level of P-gp has rarely been examined. Zuluaga et al. (2012) [31] have examined the effects of DHT and DHEA and their metabolites, 3β-diol and Adiol, on cytokine-induced inflammation and P-gp expression.

The authors also observed the up-regulation of P-gp protein levels in the spinal cords of rats from all experimental groups. The up-regulation of P-gp may be related to, or even indicate a decrease in, the permeability of the BBB and the BSCB [31]. It may also represent a protective mechanism against the excessive influx of potentially neurotoxic substances into the brain in situations whereby the barrier functions in the CNS are impaired. Moreover, it may constitute a compensatory mechanism in response to alterations in other ABC cassette transporters. Recently, Zhu et al. (2016) [34] described the inhibitory effect of antiandrogen treatment (bicalutamid) on the present ABCB1 transporter system, which plays a crucial role in the formation of prostate cancer cells. Such observations may indirectly agree with our results.

Supplementation with higher doses of androgens is mostly used in combination with strength training or bodybuilding. However, there is no satisfactory rat model of strength training involving muscle masses comparable (in relative terms) to those engaged in such training in humans [19]. Therefore, the authors decided to use well-recognized endurance training model which had been employed in earlier studies by Langfort et al. [35].

Indeed, it has been reported that exercise can modulate the redox status in microvessels of the BBB, thus helping to maintain barrier integrity [14, 36, 37]. Previous studies have also demonstrated that oxidative stress, mediating changes in BBB permeability, is associated with alterations to the structure and localization of occludin [37] and ZO-1 [38]. Furthermore, the down-regulation of occludin expression under conditions of oxidative stress is more pronounced in the absence of glucose [39]. However, studies on the expression of claudins during hypoxia have provided contradictory results [40, 41]. Reports have showed that endurance training prevented the METH-induced oxidative disruption of TJ proteins in the cerebral microvessels [42].

It is well-known that exercise can either induce or attenuate inflammation, e.g. by influencing the levels of cytokines which, in turn, may affect the BBB/BSCB. Both moderate levels of aerobic training and strenuous bouts of exercise lead to the release of IL-6 from the muscles [43]. The effect of IL-8 [44], IL-10 [45], and IL-13 [46] on tight junction protein expression has also been proven. Furthermore, there is a correlation between exercise and the expression of other cytokines, e.g. endurance training may affect the serum levels of TNFα and IFNγ in men [47].

There is a growing body of evidence suggesting the increased permeability of the BBB in different conditions is associated with hyperthermia. Watson et al. (2006) [48] showed an increase in S100β concentration in human serum after intense physical exercise in a warm environment. The changes in BBB permeability during restraint and forced swim stress were reported in animal models [49, 50]. Whole body hyperthermia has been demonstrated to result from impaired BBB integrity in mice [51]. High temperatures, above 40.0°C, have destructive effects on neuronal, glial, endothelial and epithelial cells [52]. It is also known that drug-induced brain hyperthermia induces the robust leakage of the BBB/BSCB, acute glial activation, and an increased water content, known clinically as edema [52, 53]. Altogether, these data suggest that hyperthermia, independent of its cause, significantly contributes to cell and tissue injuries in the CNS and influences barrier function. Although we did not measure the temperature of the trained rats, it could be assumed that it was increased as a result of the intense exercise.

Exercise increases the heart rate, which in turn increases the blood flow and vascular shear stress. Physical forces such as shear stress, transmural pressure, and pulsatile stretch activate the molecular mechanisms of vascular adaptation to exercise [18, 54]. These, in turn, are permanently exposed to such forces, a factor which is intensified during physical activity. Exposure to flow increases the RNA levels for a variety of tight junction proteins, including zonula ocludens-1 and 2 (ZO-1, ZO-2), and claudins. The same data showed an increase in the RNA and protein levels of VE-cadherin. Exposure of the BBB and BSCB endothelial cells to blood flow also induce increased gene expression of efflux transporters, including *MDR1* [55].

To the best of our knowledge, the present study remains unique, and in the available literature, there are no appropriate models for comparison. We believe that the present study may help to clarify the demarcation between reversible physiological change (adaptation) and irreversible pathological change (damage) which results in profound disturbances of the tight junction-related functions of the BSCB.

## Conclusions

1. The obtained results suggest that an excessive supply of testosterone may have an impact on both signal transduction in the endothelial cells of the CNS and the formation of tight junctions in the blood-spinal cord barrier.
2. Endurance training, as an independent stimulus, does not appear to affect the content of the endothelial proteins forming the blood-spinal cord barrier.

## Abbreviations

BBB: blood-brain barrier
BSCB: blood spinal cord barrier
CNS: central nervous system
IFNα: interferon α
IFNγ: interferon γ
P-gp: P glycoprotein
TJs: tight junctions
TNFα: tumor necrosis factor α
